# Association between serum aldehyde concentrations and metabolic syndrome in adults

**DOI:** 10.1007/s11356-023-27459-3

**Published:** 2023-05-19

**Authors:** Yanqun Ba, Qixin Guo, Anning Du, Beibei Zheng, Luyang Wang, Ying He, Yihong Guan, Yue Xin, Jinjin Shi

**Affiliations:** 1 Department of Cardiology, Affiliated Hangzhou First People’s Hospital, Zhejiang University School of Medicine, Hangzhou, China; 2 Department of Cardiology, the First Affiliated Hospital of Nanjing Medical University, Nanjing, China

**Keywords:** Metabolic syndrome, Isovaleraldehyde, Valeraldehyde, Serum aldehydes, J-shaped relationship, Aldehyde exposure

## Abstract

The relationship between aldehyde exposure and metabolic syndrome is unclear; hence, we aimed to investigate the association between serum aldehyde concentrations and metabolic syndrome. We analyzed the data of 1471 participants from the National Health and Nutrition Examination Survey enrolled from 2013 to 2014. The association of serum aldehyde concentrations with metabolic syndrome was assessed via generalized linear models as well as restricted cubic splines, and endpoint events were further analyzed. After adjusting for covariates, both moderate (odds ratio [OR] = 2.73, 95% confidence interval [CI]: 1.34–5.56) and high (OR = 2.08, 95% CI: 1.06–4.07) concentrations of isovaleraldehyde were associated with the risk of metabolic syndrome. Interestingly, although a moderate concentration of valeraldehyde was associated with the risk of metabolic syndrome (OR = 1.08, 95% CI: 0.70–1.65), a high concentration was not (OR = 0.55, 95% CI: 0.17–1.79). Restricted cubic splines revealed a non-linear association between valeraldehyde and metabolic syndrome, and threshold effect analysis revealed that the inflection point for valeraldehyde concentration was 0.7 ng/mL. The results of the subgroup analysis revealed differences in the relationship of aldehyde exposure with components of metabolic syndrome. High isovaleraldehyde concentrations may increase the risk of metabolic syndrome, and valeraldehyde demonstrated a J-shaped relationship with the risk of metabolic syndrome.

**Supplementary Information:**

The online version contains supplementary material available at 10.1007/s11356-023-27459-3.

## Introduction

Metabolic syndrome (MetS) involves a set of disorders, including but not limited to central obesity, insulin resistance, dyslipidemia, and hypertension. MetS is a critical risk factor contributing to the high morbidity rates of cardiovascular diseases, type 2 diabetes mellitus (DM), and cancer worldwide Aguilar et al. 2015. The prevalence of MetS in the USA, among adults aged 20 years or above, was 34% from 1999 to 2006, 33% from 2007 to 2012, and 34.7% from 2011 to 2016 (Buzzetti et al. 2016; Camacho et al. 2015; Dinkova-Kostova et al. 2002). Although the overall prevalence did not rise significantly, it is worth noting that an increase in prevalence was observed among adults aged 20–39 years, from 16.2% in 2011–2012 to 21.3% in 2015–2016. Among adults aged at least 60 years, the prevalence was 48.6% from 2011 to 2016, which was much higher than the 19.5% prevalence among adults aged 20–39 years in the same period Dinkova-Kostova et al. 2002. In view of the increasing size and age of the global population, MetS has become a public health problem worthy of attention.

MetS has a complex pathology. Previous studies have identified poor lifestyles and genetic susceptibility as major etiological factors Furukawa et al. 2004. Mounting evidence suggests that environmental factors, such as smoking and exposure to polycyclic aromatic hydrocarbons González 2022, ambient particulate matter Grundy 2016, persistent organic pollutants Grundy et al. 2005, heavy metals Haque and Ansari 2019, pesticides Hirode and Wong 2020, and noise Hotamisligil 2006, may also contribute to the development of MetS.

Aldehydes are abundant in the environment and are closely related to people’s health. Sources of human aldehyde exposure include air pollution, smoking (both tobacco cigarettes and e-cigarettes), pyrolysis of organic matter, paints, food additives, alcohol consumption, water disinfection byproducts formed via ozonation, cosmetics, hand sanitizers, and endogenous processes (Hutcheson and Rocic 2012; Kopp et al. 2003). Different aldehydes appear to have distinct effects on human health. Some aldehydes are reportedly harmful, for example, formaldehyde, crotonaldehyde, and hexanal, which are toxic and carcinogenic (Lamat et al. 2022; Li et al. 2015); others, such as cuminaldehyde and cinnamaldehyde, are beneficial and protective against obesity, hyperglycemic states, and non-alcoholic fatty liver disease (Liao et al. 2020a, b), factors commonly associated with MetS. However, in the few studies conducted on the direct relationship between aldehyde exposure and MetS, malondialdehyde levels were significantly elevated in the blood of patients with MetS, and higher plasma malondialdehyde levels were linked with a higher prevalence of MetS (McMahon et al. 2010; Moreto et al. 2014), but the underlying mechanism and causal relationship between malondialdehyde and MetS are not fully understood. Moreover, it is unknown whether other aldehydes have such an effect on MetS.

Oxidative stress and inflammation are thought to be the important mechanisms involved in the development of MetS. An endogenous aldehyde, 4-hydroxy-trans-2-nonenal (HNE), triggered by lipid peroxidation, plays a complex role in the regulation of the oxidative stress cascade and inflammatory response Mozumdar and Liguori 2011. The inhibitory effects of cuminaldehyde on non-alcoholic fatty liver disease and obesity are achieved via the alleviation of hepatic oxidative damage and hyperlipidemia Liao et al. 2020b. The above indicates that aldehydes might act as regulators of oxidative stress and inflammation and be involved in the pathogenesis of MetS. However, the possible association between aldehydes and MetS warrants further investigation. Hence, with this study, we aimed to explore the potential relationship between serum aldehyde levels and MetS based on data from the United States National Health and Nutrition Examination Survey collected from 2013 to 2014.

## Methods

### Study design and participants

The National Health and Nutrition Examination Survey is a large-scale, multi-stage, nationally representative survey of the ambulatory population in the USA, designed to assess participants’ health and nutritional status. Interview data, physical examination data, and laboratory results are collected simultaneously and published every 2 years. All data and materials are publicly available on the National Center for Health Statistics website (https://www.cdc.gov/nchs/nhanes/index.htm). Ethical approval was obtained from the National Center for Health Statistics Ethics Review Board, and all procedures were performed in accordance with the standards of the Declaration of Helsinki.

**Fig. 1:**
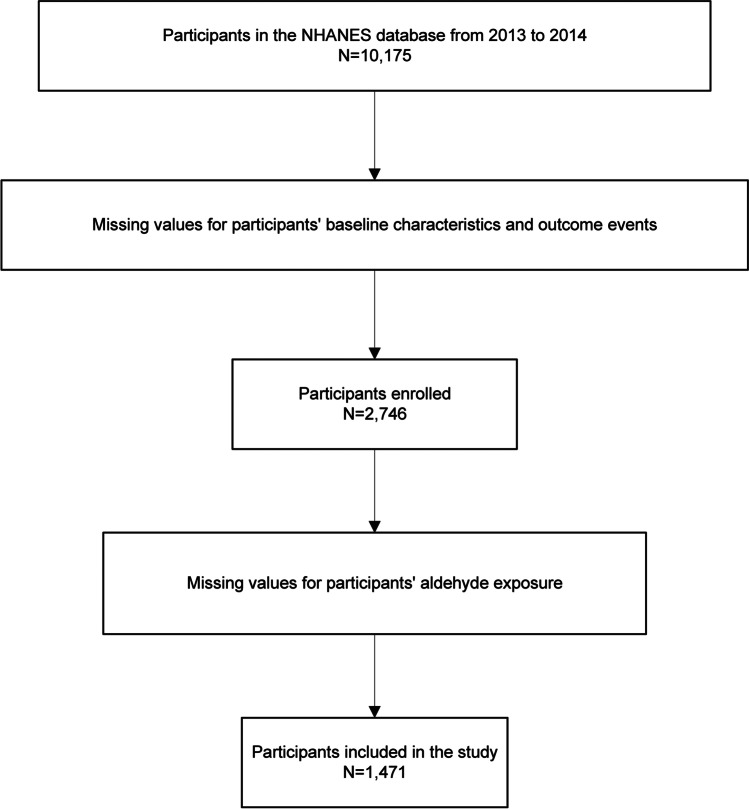
Flow chart of the study. NHANES, National Health and Nutrition Examination Survey

This continuous cross-sectional study initially included 10,175 participants enrolled from 2013 to 2014, who were aged ≥ 18 years and for whom data on aldehyde exposure were available in the National Health and Nutrition Examination Survey database. We excluded participants for whom data on the primary and secondary endpoints of this study were missing, to avoid an offset caused by data interpolation. We further excluded participants for whom data on aldehyde exposure were missing. The final analytical cohort contained 1471 participants. The entire data integration process is illustrated in Fig. 1.

### Measurement and evaluation of aldehydes

The serum levels of 12 aldehydes were determined by automated solid-phase microextraction gas chromatography and high-resolution mass spectrometry through selected ion mass detection and isotope dilution techniques Hutcheson and Rocic 2012. The limits of detection of the serum aldehydes are listed in Online Resource 1. We analyzed aldehydes with more than 75% detection frequency, including crotonaldehyde, formaldehyde, propionaldehyde, butyraldehyde, valeraldehyde, and isovaleraldehyde.

### Definitions of study outcomes and variables

Definitions of primary endpoint events were established according to the National Cholesterol Education Program’s Adult Treatment Panel III criteria for MetS Ning et al. 2021. MetS was diagnosed if three or more of the following conditions were present: high waist circumference (men: ≥ 102 cm, women: ≥ 88 cm), low levels of high-density lipoprotein cholesterol (men: < 40 mg/dL, women: < 50 mg/dL), elevated levels of triglycerides (≥ 150 mg/dL), dysglycemia (fasting blood glucose level: ≥ 110 mg/dL), high blood pressure (≥ 130/85 mmHg or using antihypertensive drugs). The secondary endpoints were a body mass index (BMI) > 30 kg/m^2^, hypertension, hyperlipidemia, and DM. Patients were considered hypertensive if they were diagnosed with hypertension by a physician, they were using antihypertensive medication, or their blood pressure was > 140/90 mmHg. Patients were diagnosed with DM if they had an elevated blood glucose level (≥ 200 mg/dL according to the 2-h oral glucose tolerance test), a fasting glucose level ≥ 126 mg/dL, a random blood glucose level ≥ 200 mg/dL, and/or a glycated hemoglobin level > 6.5%, DM was diagnosed by a physician, and/or they were using diabetes medication or insulin. Dyslipidemia was defined as a triglyceride level > 150 mg/dL, low-density lipoprotein level > 130 mg/dL, high-density lipoprotein level < 40 mg/dL, total cholesterol level > 150 mg/dL, or the use of ester-lowering drugs.

### Covariates

Covariates that might be associated with aldehyde exposure and MetS were selected based on previous studies (O'Brien et al. 2005; O'Neill and O'Driscoll 2015; Park et al. 2013; Rkhaya et al. 2018): age, sex, race/ethnicity (Mexican American, other Hispanic, non-Hispanic white, non-Hispanic black, and other races), educational level (lower than 9th grade, 9th to 11th grade, high school graduate, some college or AA degree, college graduate or above), BMI, waist circumference, energy intake, physical activity, routine blood test results, biochemical examination results of blood components, smoking habit (never, former, current), and alcohol use (current heavy alcohol user: ≥ 3 drinks per day for women, ≥ 4 drinks per day for men, or binge drinking for ≥ 5 days per month; current moderate alcohol user: ≥ 2 drinks per day for women, ≥ 3 drinks per day for men, or binge drinking for ≥ 2 days per month; current mild alcohol user: patients who did not meet the aforementioned criteria). In addition, the dietary inflammatory index, which is considered an important factor in MetS, was also included in our study.

### Statistical analyses

Continuous variables are expressed as means (standard deviations) or medians (quartiles) and categorical variables as numbers (percentages). Comparisons between groups of continuous variables, depending on normality and chi-squaredness, were performed via Welch’s *t*-test, Student’s *t*-test, and the Mann–Whitney *U* test. Comparisons of rates between the two groups were performed via the chi-square and Kruskal–Wallis *H* test for nominal and ordinal data, respectively.

Aldehydes were categorized into tertile groups, with those in the lowest tertile serving as the reference group. Logistic regression analysis was performed to assess the association between aldehyde exposure and MetS. The stability of the results was verified by including different combinations of the variables: model 1 (adjusted for age, sex, race, physical activity, and smoking status), model 2 (adjusted for age, sex, race, physical activity, smoking status, educational level, and alcohol use), and model 3 (adjusted for age, sex, race, physical activity, smoking status, educational level, alcohol use, DM, hypertension, hyperlipidemia, and BMI). The interaction between each type of aldehyde and the secondary endpoint event was analyzed, and sensitivity analysis was performed for each subgroup. Trend tests across exposure groups were performed by entering the categorical variables as continuous terms into the logistic model.

Restricted cubic splines (with nodes at the 5th, 35th, 65th, and 95th percentiles) were used to determine whether there was potential nonlinearity between aldehydes and MetS. If a nonlinear relationship was detected, a two-segment logistic regression model was applied to determine the corresponding inflection point, and the superiority of the segmented model over the single-segment model was verified via the likelihood ratio test. If the relationship was linear, the logistic model was used to determine the correlation. All statistical analyses were performed using the R software (version 4.2.0), and two-sided *p*-values < 0.05 indicated statistical significance.

## Results

### Demographic characteristics

Table 1 summarizes the baseline characteristics of aldehyde-exposed participants. A total of 1471 participants were included in this study, among whom the overall prevalence of MetS was 12.5%. Healthy participants were typically younger, had a lower BMI, glycated hemoglobin level, dietary inflammatory index, and co-occurrence of chronic diseases, and had a higher high-density lipoprotein level, energy intake, severity of alcohol abuse, and educational attainment than did patients with MetS. No statistically significant differences were observed between the groups for the other variables. Table 2 reveals that there were no significant differences in the proportions of the six aldehydes between the MetS and no-MetS groups, with a minimum missing data proportion of 19.5% and a maximum of 25.0%.

**Table 1:** Characteristics of the participants

Basic information	Total	No metabolic syndrome	Metabolic syndrome	*P*
Sex (*n,* %)	1471	1287	184	0.99
Male	733 (49.8)	641 (49.8)	92 (50.0)	
Female	738 (50.2)	646 (50.2)	92 (50.0)	
Age (years)	43 ± 19	43 ± 19	45 ± 22	< 0.0001
BMI (kg/m^2^)	28.3 ± 7.10	28.1 ± 6.95	30.0 ± 7.63	0.04
Waist circumference (cm)	97.1 ± 17.6	96.3 ± 17.0	102 ± 20.1	0.001
Race (*n*, %)				0.21
White	580 (39.4)	512 (39.8)	68 (37)	
Black	290 (19.7)	248 (19.3)	42 (22.8)	
Mexican	256 (17.4)	216 (16.8)	40 (21.7)	
Other Hispanic	145 (9.9)	131 (10.2)	14 (7.6)	
Other race	200 (13.6)	180 (14.0)	20 (10.9)	
Smoking status (*n*, %)				0.784
Never	592 (40.2)	534 (41.5)	58 (31.5)	
Former	229 (15.6)	206 (16.0)	23 (12.5)	
Current	488 (33.2)	434 (33.7)	54 (29.3)	
Unknown	162 (11.0)	113 (8.8)	49 (26.6)	
Drinking (*n*, %)				< 0.0001
Mild	373 (25.4)	333 (25.9)	40 (21.7)	
Moderate	247 (16.8)	225 (17.5)	22 (12.0)	
Heavy	256 (17.4)	238 (18.5)	18 (9.8)	
Unknown	595 (40.4)	491 (38.2)	104 (56.5)	
Educational level (*n*, %)				< 0.0001
Lower than 9th grade	201 (13.7)	147 (11.5)	53 (28.8)	
9-11th grade	287 (19.5)	243 (18.9)	44 (23.9)	
High school graduate	303 (20.6)	271 (21.1)	32 (17.4)	
Some college or AA degree	407 (27.7)	372 (28.9)	35 (19)	
College graduate or above	273 (18.6)	253 (19.7)	20 (10.9)	
Laboratory tests				
WBC (× 10^9^/L)	7.43 ± 2.29	7.42 ± 2.29	7.47 ± 2.35	0.204
RBC (× 10^12^/L)	4.69 ± 0.463	4.68 ± 0.465	4.75 ± 0.446	0.665
Hb (mg/dL)	14.1 ± 1.51	14.1 ± 1.50	14.1 ± 1.62	0.255
PLT (× 10^9^/L)	240 ± 60.1	240 ± 58.8	240 ± 68.3	0.152
Alt (U/L)	24.7 ± 22.6	24.4 ± 22.9	26.9 ± 19.7	0.273
Ast (U/L)	25.4 ± 16.4	25.0 ± 16.1	28.3 ± 18.3	0.012
HbA1c (%)	5.63 ± 0.912	5.54 ± 0.765	6.28 ± 1.45	< 0.0001
HDL (mmol/L)	1.34 ± 0.398	1.38 ± 0.398	1.10 ± 0.309	0.002
LDL (mmol/L)	2.78 ± 0.885	2.83 ± 0.882	2.49 ± 0.848	0.319
Exercise and diet				
Physical activity	2100 (840–5410)	2080 (840–5450)	2340 (720–5250)	0.970
MET: recreational activity	1080 (540–2160)	1090 (540–2160)	1000 (520–2580)	0.773
MET: work activity	2880 (840–7680)	3000 (915–7620)	2640 (780–9660)	0.906
MET: walking and bicycle use	600 (280–1680)	670 (285–1680)	420 (200–930)	0.046
Energy (kcal)	2035 ± 857	2067 ± 887	1804 ± 660	0.002
Protein (g/d)	79.5 ± 36.5	80.4 ± 37.7	73.2 ± 29.9	0.050
Carbohydrate (g/d)	247 ± 110	250 ± 114	221 ± 81	0.002
Total sugars (g/d)	110 ± 68	112 ± 70	97 ± 52	0.023
Dietary fiber (g/d)	15.9 ± 8.49	16.1 ± 8.63	14.3 ± 7.21	0.001
Total fat (g/d)	77.4 ± 38.1	78.5 ± 38.6	69.5 ± 33.8	0.158
Dietary inflammatory index	1.024 ± 1.715	0.983 ± 1.71	1.313 ± 1.5	0.030
Disease history (*n*, %)				
Diabetes mellitus	220 (15)	140 (10.9)	80 (43.5)	< 0.0001
Hypertension	513 (34.9)	411 (31.9)	102 (55.4)	< 0.0001
Hyperlipidemia	935 (63.6)	789 (61.3)	146 (79.3)	< 0.0001

Data are presented as mean (SD), median (interquartile range), or *n* (%). *BMI*, body mass index; *Alt*, alanine transaminase; *Ast*, aspartate aminotransferase; *HbA1c*, glycated hemoglobin type A1C; *HDL*, high-density lipoprotein; *LDL*, low-density lipoprotein; *MET*, metabolic equivalent; *WBC*, white blood cell; *RBC*, red blood cell; *PLT*, platelets; *Hb*, hemoglobin

**Table 2:** Serum concentrations (ng/mL) of six aldehydes quantified in the study sample

Aldehydes	Total (ng/mL)	No-metabolic syndrome (ng/mL)	Metabolic syndrome (ng/mL)	*P*	*n* (%)	Missing data (%)
	*n* = 1471	*n* = 1287	*n* = 184			
Crotonaldehyde	0.104 (0.104–0.176)	0.104 (0.104–0.176)	0.104 (0.104–0.178)	0.841	2087 (75.5)	677 (24.5)
Formaldehyde	136 (124–143)	136 (124–149)	136 (126–148)	0.462	2224 (80.5)	540 (19.5)
Propionaldehyde	2.03 (1.54–2.59)	2.03 (1.54–2.60)	1.94 (1.53–2.49)	0.328	2063 (75)	701 (25)
Butyraldehyde	0.538 (0.374–0.705)	0.532 (0.370–0.704)	0.556 (0.421–0.705)	0.258	2085 (75.4)	679 (24.6)
Valeraldehyde	0.223 (0.223–0.402)	0.223 (0.223–0.402)	0.223 (0.223–0.392)	0.851	2108 (76.3)	656 (23.7)
Isovaleraldehyde	0.471 (0.323–0.922)	0.474 (0.326–0.930)	0.455 (0.295–0.846)	0.127	2155 (78.0)	609 (22)

Data are presented as medians (interquartile ranges)

### Relationship between aldehyde levels and primary endpoint events

**Fig. 2:**
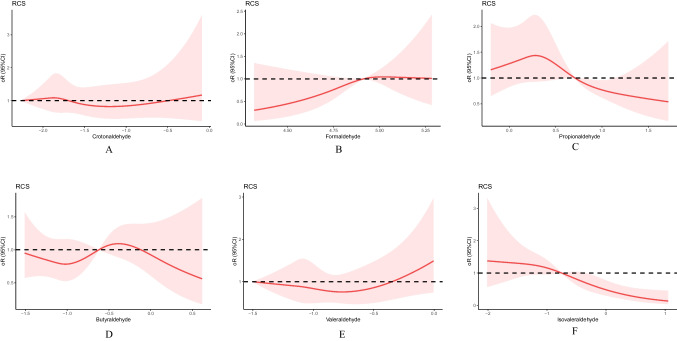
Restricted cubic splines for the association between aldehyde exposure and metabolic syndrome, adjusted for the confounders hypertension, hyperlipidemia, hyperglycemia, body mass index, waist circumference, energy use, dietary inflammatory index, high-density lipoprotein level, aspartate aminotransferase level, education level, glycated hemoglobin level, and alcohol use. Red lines and shaded areas represent the odds ratios and 95% confidence intervals, respectively. **a** Crotonaldehyde, **b** formaldehyde, **c** propionaldehyde, **d** butyraldehyde, **e** valeraldehyde, and **f** isovaleraldehyde. RCS, restricted cubic spline; OR, odds ratio; CI, confidence interval

In the fully adjusted model, the 95% confidence intervals (CIs) and odds ratios (ORs) for MetS in the second and third tertiles, respectively, compared with the lowest tertiles group were 1.14 (0.74–1.76) and 5.98 (1.78–20.16) for crotonaldehyde; 0.74 (0.45–1.20) and 0.76 (0.47–1.20) for formaldehyde; 1.77 (1.04–3.00) and 1.30 (0.77–2.19) for propionaldehyde; 0.92 (0.56–1.51) and 1.04 (0.65–1.68) for butyraldehyde; 1.08 (0.70–1.65) and 0.55 (0.17–1.79) for valeraldehyde; and 2.73 (1.34–5.56) and 2.08 (1.06–4.07) for isovaleraldehyde. Isovaleraldehyde was associated with the development of MetS as indicated by the trend test (*p* = 0.003, 0.036, and 0.008 in models 1, 2, and 3, respectively). The same was true for moderate propionaldehyde exposure (*p* for trend = 0.009, 0.038, and 0.034 in models 1, 2, and 3, respectively). Further details are presented in Table 3. The association between valeraldehyde and MetS was nonlinear (test for nonlinearity, *p* = 0.047). However, no nonlinear relation was observed for the other five aldehydes (Online Resource 2). Restricted cubic spline plots revealed a J-shaped association between valeraldehyde and MetS, and we further performed smoothed curve fitting with threshold effect analysis. The inflection point of valeraldehyde was 0.7 ng/mL. In addition, we present the relationship between exposure to the six major aldehydes and MetS, adjusted for covariates, in Fig. 2.

**Table 3:** Adjusted odds ratios for associations between aldehydes and the prevalence of metabolic syndrome

	Model 1	*p*	*P-*trend	Model 2	*p*	*P*-trend	Model 3	*p*	*P*-trend
	OR (95% CI)			OR (95% CI)			OR (95% CI)		
Crotonaldehyde			0.606			0.598			0.647
Q1	1			1			1		
Q2	1.15 (0.77–1.71)	0.502		1.16 (0.77–1.74)	0.479		1.14 (0.74–1.76)	0.549	
Q3	4.46 (1.57–12.66)	0.005		5.92 (1.96–17.89)	0.002		5.98 (1.78–20.16)	0.004	
Formaldehyde			0.256			0.231			0.209
Q1	1			1			1		
Q2	0.78 (0.50–1.21)	0.260		0.77 (0.49–1.20)	0.242		0.74 (0.45–1.20)	0.219	
Q3	0.83 (0.53–1.30)	0.390		0.77 (0.49–1.20)	0.246		0.76 (0.47–1.20)	0.258	
Propionaldehyde			0.009			0.038			0.034
Q1	1			1			1		
Q2	1.91 (1.18–3.09)	0.009		1.67 (1.02–2.74)	0.042		1.77 (1.04–3.00)	0.036	
Q3	1.46 (0.90–2.36)	0.127		1.24 (0.76–2.04)	0.386		1.30 (0.77–2.19)	0.328	
Butyraldehyde			0.810			0.572			0.75
Q1	1			1			1		
Q2	0.94 (0.60–1.49)	0.800		0.87 (0.55–1.39)	0.570		0.92 (0.56–1.51)	0.743	
Q3	1.04 (0.68–1.60)	0.860		0.99 (0.64–1.55)	0.980		1.04 (0.65–1.68)	0.868	
Valeraldehyde			0.504			0.829			0.672
Q1	1			1			1		
Q2	1.14 (0.77–1.67)	0.522		1.04 (0.70–1.54)	0.855		1.08 (0.70–1.65)	0.729	
Q3	0.84 (0.28–2.46)	0.743		0.76 (0.25–2.29)	0.622		0.55 (0.17–1.79)	0.320	
Isovaleraldehyde			0.003			0.036			0.008
Q1	1			1			1		
Q2	2.87 (1.52–5.41)	0.001		2.26 (1.18–4.35)	0.015		2.73 (1.34–5.56)	0.006	
Q3	2.29 (1.28–4.11)	0.005		2.11 (1.14–3.90)	0.017		2.08 (1.06–4.07)	0.033	

Model 1 was adjusted for age, sex, race, physical activity, and smoking status

Model 2 was adjusted for age, sex, race, physical activity, smoking status, educational level, and alcohol use

Model 3 was adjusted for age, sex, race, physical activity, smoking status, educational level, alcohol use, diabetes mellitus, hypertension, hyperlipidemia, and body mass index

OR: odds ratio, CI: confidence interval, Q: quantile

### Relationship between aldehyde levels and secondary endpoint events

The association between aldehyde levels and four common components of MetS (hypertension, hyperglycemia, hyperlipidemia, and BMI ≥ 30 kg/m^2^) was analyzed (Table 4). After adjusting for covariates, we discovered that a moderate concentration of propionaldehyde was negatively associated with the risk of developing hyperlipidemia, but not the lowest concentration (OR = 0.62, 95% CI: 0.45–0.87, *p* for trend = 0.004). Similarly, a moderate concentration of serum isovaleraldehyde was negatively associated with the risk of hyperlipidemia (OR = 0.59, 95% CI: 0.38–0.90, *p* for trend = 0.017), but not a high concentration (OR = 0.71, 95% CI: 0.48–1.07, *p* for trend = 0.017). None of the other aldehydes were associated with any components of MetS.

**Table 4:** Multivariable associations of selected aldehydes with secondary endpoint events

	Hypertension	Diabetes mellitus	Hyperlipidemia	BMI ≥ 30 kg/m^2^
	OR (95% CI)	OR (95% CI)	OR (95% CI)	OR (95% CI)
Crotonaldehyde				
Q1	1	1	1	1
Q2	1.13 (0.83–1.54)	0.83 (0.56–1.22)	0.96 (0.73–1.26)	0.86 (0.66–1.14)
Q3	1.44 (0.40–4.70)	2.09 (0.58–7.54)	1.12 (0.39–3.18)	1.21 (0.45–3.23)
*p* for trend	0.450	0.308	0.769	0.283
Formaldehyde				
Q1	1	1	1	1
Q2	1.05 (0.74–1.48)	1.04 (0.67–1.64)	1.06 (0.78–1.44)	1.18 (0.86–1.61)
Q3	1.08 (0.76–1.53)	0.95 (0.61–1.50)	1.23 (0.90–1.68)	1.04 (0.76–1.43)
*p* for trend	0.797	0.859	0.719	0.307
Propionaldehyde				
Q1	1	1	1	1
Q2	0.87 (0.60–1.26)	1.23 (0.76–1.99)	0.62 (0.45–0.87)	1.03 (0.74–1.43)
Q3	0.77 (0.54–1.11)	0.96 (0.60–1.54)	0.94 (0.68–1.30)	0.88 (0.64–1.23)
*p* for trend	0.451	0.403	0.004	0.817
Butyraldehyde				
Q1	1	1	1	1
Q2	0.93 (0.65–1.33)	0.78 (0.49–1.25)	0.84 (0.61–1.15)	0.79 (0.57–1.09)
Q3	0.88 (0.62–1.23)	1.02 (0.66–1.57)	0.81 (0.60–1.11)	0.97 (0.71–1.31)
*p* for trend	0.677	0.315	0.270	0.154
Valeraldehyde				
Q1	1	1	1	1
Q2	0.93 (0.68–1.26)	1.03 (0.69–1.53)	0.86 (0.65–1.12)	0.99 (0.75–1.30)
Q3	1.41 (0.64–3.11)	1.65 (0.69–3.96)	1.06 (0.51–2.17)	0.80 (0.38–1.65)
*p* for trend	0.583	0.965	0.253	0.964
Isovaleraldehyde				
Q1	1	1	1	1
Q2	1.12 (0.69–1.81)	0.69 (0.36–1.32)	0.59 (0.38–0.90)	0.97 (0.64–1.49)
Q3	1.15 (0.74–1.79)	1.48 (0.85–2.59)	0.71 (0.48–1.07)	1.03 (0.69–1.54)
*p* for trend	0.714	0.095	0.017	0.840

The model was adjusted for age, sex, race, physical activity, smoking status, educational level, and alcohol use

*OR*, odds ratio; *CI*, confidence interval; *BMI*, body mass index; *Q*, quantile

### Subgroup analysis

**Fig. 3:**
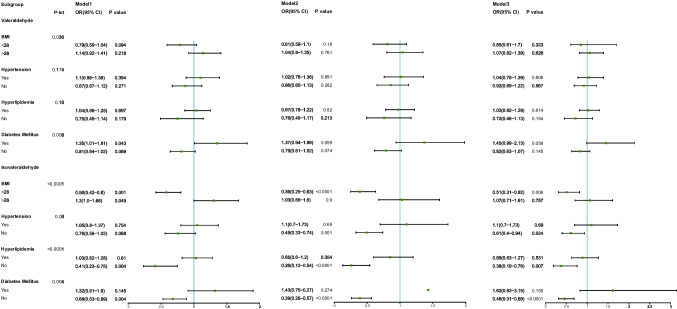
Subgroup analysis of the associations of valeraldehyde and isovaleraldehyde concentrations with metabolic syndrome. Model 1 was adjusted for age and sex. Model 2 was adjusted for age, sex, race, physical activity, and smoking status. Model 3 was adjusted for age, sex, race, physical activity, smoking status, educational level, and alcohol use. *P*-trend, *p* for trend; p-int, *p* for interaction; OR, odds ratio; CI, confidence interval, BMI, body mass index

The subgroup analysis showed significant interactions between isovaleraldehyde and obesity, hyperglycemia, hypertension, and hyperlipidemia, with the overall benefits of altering blood aldehyde concentrations after fully adjusting for variables being 0.51 (0.31–0.82) in the non-obese, 0.61 (0.40–0.94) in the non-hypertensive, 0.38 (0.19–0.76) in the non-hyperlipidemic, and 0.46 (0.31–0.69) in the non-diabetic populations, which was consistent across models for most of the components (p < 0.05 for all three models, except for hypertension in model 1). In addition, strong interactions were observed between valeraldehyde and obesity and DM. However, these results were not stable in the sensitivity analysis; for example, for the diabetic population, different results were reported when different covariates were included (*p* = 0.043, 0.099, and 0.058 in models 1, 2, and 3, respectively). No statistically significant associations were observed for the other strata (Fig. 3).

## Discussion

The pathogenesis of MetS is not fully understood. Few studies have been conducted on the relationship between aldehyde exposure and the MetS risk. To our knowledge, this is one of the first studies to explore the association between levels of individual serum aldehydes and MetS and its subtypes. After adjusting for several variables, including age, sex, race, physical activity, smoking habit, educational level, alcohol use, DM, hypertension, hyperlipidemia, and BMI, isovaleraldehyde was associated with the development of MetS as shown by the trend test. Interestingly, a J-shaped relationship was observed between valeraldehyde exposure and MetS based on the segmented model, with an inflection point of 0.7 ng/mL, which was not observed for the other five aldehydes.

The mechanisms leading to the development of MetS are complex; however, oxidative stress and inflammatory responses are the leading causes (Selcuk et al. 2012; Silva et al. 2018). Obesity and insulin resistance lead to elevated levels of systemic oxidative stress in patients with MetS. Increased oxidative stress caused by accumulated fat, accompanied by an augmented expression of NADPH oxidase and decreased expression of antioxidative enzymes (causing dysregulation of plasminogen activator inhibitor-1, TNF-α, resistin, leptin, adiponectin, and other adipocytokines), promotes the development of MetS Sinharoy et al. 2019. Systemic oxidative stress further activates the downstream inflammatory cascade, significantly increasing levels of inflammatory markers, such as IL6, CRP, and TNF-α Sonowal and Ramana 2019. Persistent chronic inflammation exacerbates the progression of cardiovascular diseases and DM Timucin and Basaga 2017. Several studies have demonstrated a relationship between aldehyde levels and oxidative stress and inflammation. In a state of insulin resistance, acetaldehyde levels in smooth muscle cells may be upregulated, which induces oxidative stress and promotes vasoconstriction, leading to hypertension, whereas the inhibition of acetaldehyde accumulation may prevent hypertension Vasdev et al. 2004. Furthermore, in rats, cuminaldehyde reportedly improves high-fat diet-induced non-alcoholic fatty liver disease, a key component of MetS, by lowering oxidative stress and hyperlipidemia (Liao et al. 2020b; Weng et al. 2022). HNE, the most abundant and toxic lipid peroxidation end product, reportedly regulates both oxidative stress and inflammation. It induces cellular antioxidant defense via the KEAP1-Nrf2 signaling pathway (Mozumdar and Liguori 2011; Xu et al. 2021; Xue et al. 2008). Interestingly, HNE can either activate or inhibit NFκB signaling to regulate various proinflammatory pathways in a concentration-dependent manner; a low concentration can activate NFκB, while a high concentration acts as an inhibitor thereof (Mozumdar and Liguori 2011; Zhang et al. 2020; Zhang et al. 2021; Zhu et al. 2022). In this study, we discovered that isovaleraldehyde and valeraldehyde were closely associated with MetS, likely related to the regulation of oxidative stress and inflammation. However, our results need to be validated in further animal experiments.

In this study, isovaleraldehyde was inversely associated with MetS and valeraldehyde displayed a J-shaped relationship with MetS, which suggests that a higher serum concentration of isovaleraldehyde may be protective against MetS, and a low dose (less than 0.7 ng/mL) may also have beneficial effects. As these two aldehydes are not known to be teratogenic, carcinogenic, or potentially harmful to human beings at such a concentration, they may provide promising avenues for the treatment of MetS.

Few large studies have been conducted to explore the relationship between aldehyde exposure and MetS. Notably, recent population-based studies revealed that benzaldehyde and isopentanaldehyde are strongly related to obesity and cardiovascular diseases, highlighting the crucial role of aldehyde exposure for metabolic regulation in humans (O'Brien et al. 2005; O'Neill and O'Driscoll 2015). However, those studies did not further elucidate the underlying association between aldehydes and MetS. This is, to our knowledge, the first large-scale, nationwide population database study in which the relationship between aldehyde exposure and MetS was examined. We improved the accuracy and reliability of the results by excluding aldehydes for which more than 25% of the data were missing. Multiple adjustments on the data were performed, and different models were established to analyze potential relationships, both qualitatively and quantitatively. However, this study also has some limitations. First, aldehydes are known to have a wide range of sources. The serum aldehyde data that we used in this study reflect the overall exposure of each aldehyde and cannot be used to accurately distinguish between exogenous and endogenous sources. Second, the cross-sectional study design prevented the exclusion of reverse causality. Therefore, in the future, a longitudinal study is required to overcome this limitation. Third, the biological effects (the underlying mechanism) of valeraldehyde and isovaleraldehyde on the development of MetS need to be further validated through animal experiments. Fourth, the participants in this study were from a single geographical location; thus, the generalizability of the conclusions to other populations remains to be confirmed. For a better understanding of the effect of aldehyde exposure on human health and homeostasis, the normal serum concentration ranges of different aldehydes need to be established.

## Conclusions

Isovaleraldehyde and valeraldehyde concentrations are associated with MetS, with the latter having a J-shaped relationship. Further studies are necessary to clarify causality and reveal potential underlying mechanisms of action.

## Supplementary Information

Below is the link to the electronic supplementary material.

Supplementary file1 (DOCX 160 KB)

## Data Availability

The data used in this paper can be obtained from the NHANES database.
