# Supplementary material for: Association between serum aldehyde concentrations and metabolic syndrome in adults
Source: Environ Sci Pollut Res Int. 2023 May 19;30(29):74290–300. doi: 10.1007/s11356-023-27459-3 (PMC10287802; doi:10.1007/s11356-023-27459-3)
Supplement: Supplementary file 1 — Supplementary file1 (DOCX 160 KB) [file 11356_2023_27459_MOESM1_ESM.docx]

Supplementary Information

Association between serum aldehyde concentrations and metabolic syndrome in adults

*Environmental Science and Pollution Research*

Yanqun Ba, Qixin Guo, Anning Du, Beibei Zheng, Luyang Wang, Ying He, Yihong Guan, Yue Xin, Jinjin Shi

*** Correspondence:** Qixin Guo: Guoqixin@stu.njmu.edu.cn

**Online Resource 1.** Limits of detection and reference ranges of serum aldehydes

| Serum aldehydes (ng/mL) | LOD | Above LOD (%) |
| --- | --- | --- |
| Benzaldehyde | 0.461 | 91.8 |
| Butyraldehyde | 0.313 | 82.0 |
| Crotonaldehyde | 0.147 | 36.9 |
| Decanaldehyde | 3.9 | 0.7 |
| Heptanaldehyde | 0.312 | 91.8 |
| Hexanaldehyde | 0.693 | 97.5 |
| Isovaleraldehyde | 0.119 | 99.2 |
| Nonanaldehyde | 2.63 | 38.7 |
| Octanaldehyde | 0.66 | 15.0 |
| Formaldehyde | 0.142 | 3.9 |
| Valeraldehyde | 0.119 | 35.2 |
| Propionaldehyde | 1.16 | 90.9 |

LOD, limit of detection


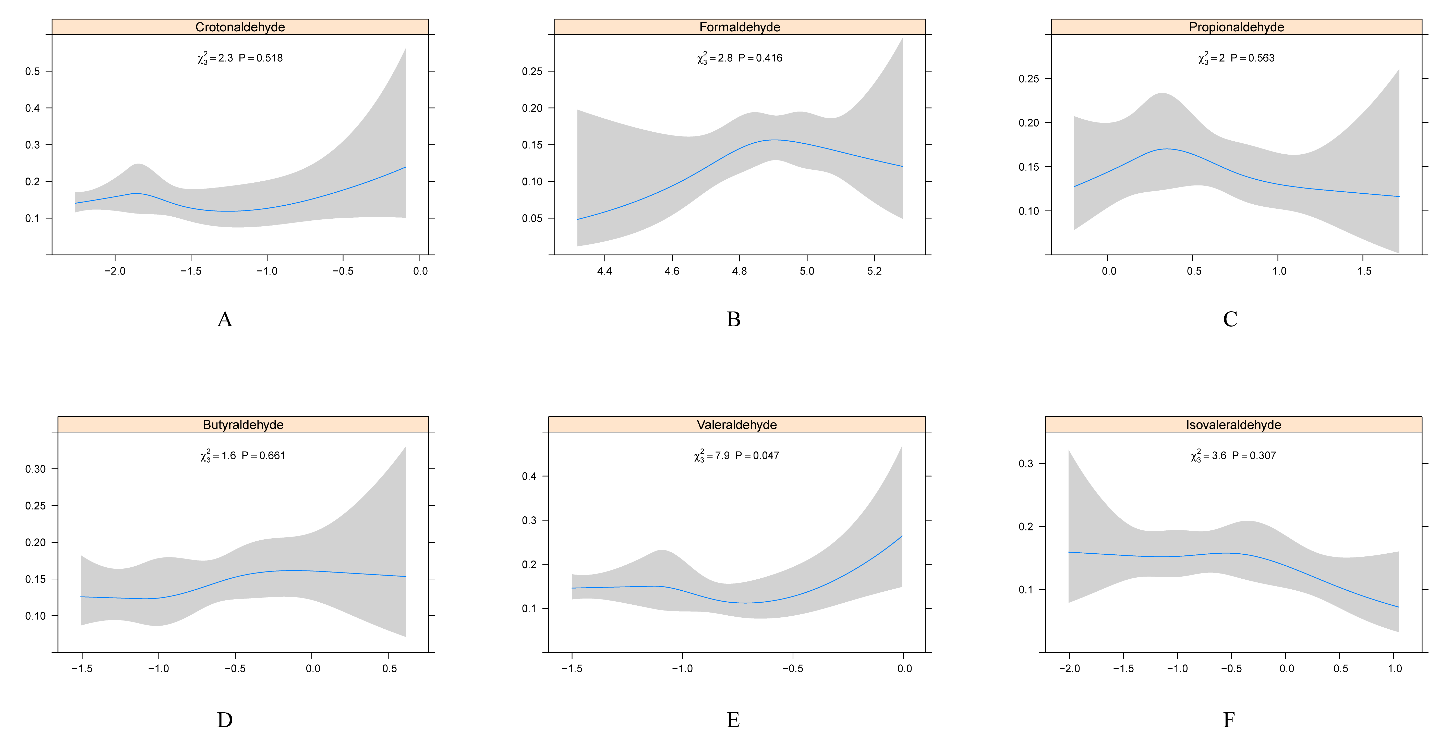


**Online Resource 2.** The linear relationship between aldehyde exposure and metabolic syndrome. P <0.05 indicates a nonlinear relationship. (**A**) Crotonaldehyde, (**B**) formaldehyde, (**C**) propionaldehyde, (**D**) butyraldehyde, (**E**) valeraldehyde, and (**E**) isovaleraldehyde
